# Impact of anaemia on the prognosis of myocardial infarction in black Africans

**Published:** 2009-08

**Authors:** C Konin, M Adoh, J Koffi, JB Anzouan-Kacou, A Adoubi, E Kramoh, E Ake-Traboulsy

**Affiliations:** Cardiology Institute of Abidjan and University of Cocody, Abidjan, Ivory Coast; Cardiology Institute of Abidjan and University of Cocody, Abidjan, Ivory Coast; Cardiology Institute of Abidjan and University of Cocody, Abidjan, Ivory Coast; Cardiology Institute of Abidjan and University of Cocody, Abidjan, Ivory Coast; Cardiology Institute of Abidjan and University of Cocody, Abidjan, Ivory Coast; Cardiology Institute of Abidjan and University of Cocody, Abidjan, Ivory Coast; Cardiology Institute of Abidjan and University of Cocody, Abidjan, Ivory Coast

## Abstract

**Objective:**

Anaemia is increasingly being described as a negative predictor of outcome after myocardial infarction. The objective of our study was to assess the prognosis post myocardial infarction in the short and medium term in black Africans with chronic anaemia.

**Methods:**

We carried out a comparative case-control study on 272 patients (93 anaemic and 179 non-anaemic) hospitalised for myocardial infarction at the Cardiology Institute of Abidjan. One group included 93 patients who presented with anaemia concurrent with the myocardial infarction (haemoglobin level low: 13 g/dl for males and 12 g/dl for females, respectively). The other group comprised 172 patients who presented without anaemia during the acute phase of myocardial infarction. The haemoglobin rate was measured at admission, as were the biological markers of myocardial infarction.

**Results:**

The mean age was 53.5 years for the anaemic patients and 52.6 years for the non-anaemic patients. We noticed a clear male predominance in both populations (81.7 vs 78.8%; *p* = 0.56). The mean haemoglobin level was lower in the anaemic patients compared to that in the non-anaemic patients (10.2 vs 15 g/dl). The anaemic patients were eight times more at risk for an unfavourable outcome (complications or death) compared to the non-anaemic patients (91.4 vs 57%; OR = 8.02; 95% CI: 3.5–19.07; chi^2^ = 33.74; *p* < 0.0001). The anaemic patients were 3.7 times more at risk for right ventricular failure (NYHA class II and III) compared to the control population (69.9 vs 38.5%; OR = 3.7; 95% CI: 08–6.60; chi^2^ = 24.06; *p* < 0.0001) and six times more at risk for cardiogenic shock (24.7 vs 5.3%; OR = 6.21; 95% CI: 2.56–15.43; chi^2^ = 22.89; *p* < 0.0001). The mortality rate was significantly higher in the anaemic than the non-anaemic patients (35.5 vs 12.8%; OR = 3.73; 95% CI: 1.94–7.19; chi^2^ = 19.18; *p* < 0.0001).

**Conclusion:**

Anaemia is an independent risk factor for a poor prognosis during the acute phase of myocardial infarction in black Africans.

## Abstract (fr)

**But:**

L’anémie est de plus en plus décrite comme un facteur de pronostic péjoratif de l’infarctus du myocarde. Le but était d’évaluer le pronostic à court et moyen terme de l’infarctus du myocarde chez le Noir Africain porteur d’une anémie chronique.

**Methode:**

Nous avons effectué une étude rétrospective comparative concernant 272 patients (93 anémiés et 179 nonanémiés) hospitalisés à l’Institut de Cardiologie d’Abidjan pour infarctus du myocarde. Le premier groupe était constitué de patients qui ont présenté concomitamment à l’infarctus du myocarde une anémie (taux d’hémoglobine inférieur respectivement à 13 g/dl pour le sexe masculin et 12 g/dl pour le sexe féminin). Ils étaient au nombre de 93. Le deuxième groupe était constitué de patients qui n’ont pas présenté d’anémie à la phase aigue de l’infarctus du myocarde. Ils étaient au nombre de 179. Le taux d’hémoglobine a été dosé à l’inclusion en même temps que les marqueurs biologiques de l’infarctus du myocarde.

**Resultats:**

La moyenne d’âge était de 53.5 ans pour les anémiés et 52.6 ans pour les non anémiés. Nous avons observé une nette prédominance masculine dans l’ensemble des deux populations (81.7 vs 78.8%; *p* = 0.56). La moyenne du taux d’hémoglobine était plus basse chez les anémiés par rapport aux non anémiés (10.2 vs 15 g/dl)). Les anémiés ont eu huit fois plus de risque d’avoir une évolution défavorable (complication ou décès) par rapport aux non anémiés (91.4 vs 57%; OR = 8.02; IC 95%: 3.5–19.07; Khi^2^ = 33.74; *p* < 0.0001).

Les anémiés ont eu 3.7 plus de risque de présenter une insuffisance ventriculaire gauche (KILLIP II et III) par rapport aux témoins (69.9 vs 38.5%; OR = 3.7; IC 95%: 2.08–6.60; Khi^2^ = 24.06; *p* < 0.0001) et six fois plus de risque pour le choc cardiogénique (24.7 vs 5.3%; OR = 6.21; IC 95%: 2.56–15.43; Khi^2^ = 22.89; *p* < 0.0001). La mortalité a été significativement plus élevée chez les anémiés que chez les non anémiés (35.5 vs 12.8%; OR = 3.73; IC 95%: 1.94–7.19; Khi^2^ = 19.18; *p* < 0,0001).

**Conclusion:**

l’anémie a représenté un facteur indépendant de mauvais pronostic à la phase aiguë de l’infarctus du myocarde chez le noir Africain.

## Resumé

L’anémie est de plus en plus décrite comme un facteur de mauvais pronostic au cours de l’infarctus du myocarde. Il est prouvé qu’elle constitue un facteur indépendant de surmortalité à la phase aigue de cette coronaropathie aigue.^1^-^3^ Mais ce rôle délétère de l’anémie ne fait pas l’unanimité. En effet Al Faaluji^4^ dans sa série n’a trouvé aucune relation directement significative de l’anémie sur la mortalité chez les patients atteints d’infarctus du myocarde.

Devant ces controverses quant au rôle pronostique réel de l’anémie au cours de l’infarctus du myocarde, et devant la prévalence élevée de l’anémie en Afrique subsaharienne, mais surtout devant la mortalité post infarctus encore élevée dans cette région, nous avons initié cette étude comparative. Elle avait pour but d’évaluer le pronostic à court et moyen terme de l’infarctus du myocarde chez le Noir Africain porteur d’une anémie chronique.

## Methodologie

Nous avons effectué une étude rétrospective comparative concernant deux groupes de patients hospitalisés à l’Institut de Cardiologie d’Abidjan pour infarctus du myocarde aigu, dont la douleur évolue depuis moins de 24 heures. Le premier groupe était constitué de 93 patients qui ont présenté à l’admission une anémie concomitamment à leur infarctus du myocarde. Le deuxième groupe était constitué de 179 patients hospitalisés durant la même période pour infarctus du myocarde et qui n’avaient pas présenté d’anémie. Il s’agissait d’une étude cas-témoins (un cas/deux témoins) où les cas étaient représentés par les anémiés et les témoins représentés par les non anémiés.

L’appariement des deux populations s’est effectué selon l’age, le sexe, le siège de la nécrose et les facteurs de risque cardiovasculaires majeurs (HTA, tabac, diabète, obésité, hypercholestérolémie). Les critéres de jugement dans chaque groupe de population étaient représentés par les éventuelles complications hémodynamiques, rythmiques, conductives ainsi que la mortalité à la phase aigue de l’infarctus du myocarde.

Le diagnostic de l’infarctus du myocarde était basé sur les critères cliniques (syndrome coronarien aigu résistant à la trinitrine), électrocardiographiques (onde de Pardee, onde Q), biologiques (élévation des enzymes cardiaques, élévation de la troponine I).

La définition de l’anémie a été faite selon les critères de l’OMS,^5^ à savoir:

● Le taux d’hémoglobine inférieur à 12 g/dl et l’hématocrite inférieur à 36% chez la femme.
● Le taux d’hémoglobine inférieur à 13 g/dl et l’hématocrite inférieur à 39% chez l’homme.

L’hémogramme a été effectué aux urgences dès l’admission des patients en même temps que les marqueurs biologiques avant toute thérapeutique. Tous les patients ont bénéficié d’un traitement médical à base d’anticoagulant (héparine), inhibiteur de l’enzyme de conversion, bêtabloquant, dérivés nitrés, antiagrégant plaquettaire. Six patients de chaque groupe ont bénéficié de la thrombolyse avant la sixème heure par Streptokinase. Aucun patient n’a bénéficié de l’angioplastie. Ont été exclus de l’étude les patients admis pour syndrome coronarien aigu sans sus décalage de ST et sans modification enzymatique. Ont été également exclus les patients porteurs d’un infarctus du myocarde dont la douleur évoluait depuis plus de 24 heures. Le sous groupe des patients de plus de 65 ans a fait l’objet de la même étude avec comme critéres de jugement les complications et la mortalité à la phase aigue de l’infarctus du myocarde.

L’analyse statistique des données a été réalisée grâce au logiciel Epi Info 6 version 6.04. La comparaison des deux populations a été effectué grâce au calcul du rapport des côtes (odds ratio), confirmé par le test de Khi2 au seuil 5%. Les effectifs inférieurs à 5 ont été comparés grâce au test de Fisher au seuil 5%.

## Resultats

La moyenne d’âge était de 53.5 ans pour les anémiés et 52.6 ans pour les non anémiés. Nous avons observé une nette prédominance masculine dans l’ensemble des deux populations (81.7 vs 78.8%; *p* = 0.56). La moyenne du taux d’hémoglobine était de 10.2 g/dl chez les anémiés (extrêmes 5.3 g/dl et 11.9 g/dl. Vingt d’entre eux (21.5%) avaient un taux d’hémoglobine inférieur à 7 g/dl, et 38.7% un taux d’hémoglobine compris 7 et 10 g/dl. La moyenne du taux d’hémoglobine était comprise entre 10 et 12.5 g/dl dans 39.8%. Quant aux non anémiés la moyenne du taux d’hémoglobine était de 15g/dl (extrêmes 12.7 g/dl et 17 g/dl.

La moyenne de la durée d’hospitalisation était de 21 jours (extrêmes un et 82 jours) chez les anémiés, et 19.4 jours (extrêmes un et 89 jours) chez les témoins (*p* = 0.03).Une prédominance du siège antérieure a été observée dans les deux groupes (71 vs 75%; *p* = 0.49)

Le tabac a été le facteur de risque le plus fréquent dans les deux populations (59 vs 59.8%; *p* = 0.91). Le taux moyen d’intoxication tabagique était de 26 paquet-années. L’hypertension artérielle est au second rang (55.9 vs 59.7%; *p* = 0.38); suivie de l’hypercholestérolémie (46.2 vs 48.6%; *p* = 0.14), du diabète (31.2 vs 34.1%, *p* = 0.63) et de l’obésité (24.7 vs 25.1%; *p* = 0.94). L’indice de masse corporel a été en moyenne 25.5 kg/m^2^ chez les anémiés et 26.4 kg/m^2^ chez les non anémiés.

La ménopause a été retrouvée chez 58.3% des femmes. Les hémoglobinopathies ont été observées chez 12 patients et réparties comme suit: six cas d’hémoglobine AS, quatre hémoglobine AC et deux bétathalassémies. Les porteurs d’hémoglobinopathies AC et les bétathalassémies étaient anémiés, les AS ne l’étaient pas. Seuls sept patients du groupe des anémiés qui avaient un taux d’hémoglobine inférieur à 7 g/dl avec des signes d’intolérance ont bénéficié d’une transfusion sanguine.

Les caractéristiques sociodémographiques et cliniques initiales des deux populations sont comparées dans le tableau 1.

**Tableau 1.** Caracteristiques Initiales De Nos Populations D’etude (Initial Characteristics Of Our Study Population)

*Variables*	*Anémiés (n = 93)*	*Non-anémies (n = 179)*	*OR*	*IC 95%*	*Khi^2^*	*p*
Age (ans)	53.5	52.6	–	–	–	NS
Sexe (M/F)	76/17	141/38	0.83	(0.42–1.64)	0.33	NS
Taux d’Hg (g/dl)	10.2	15				NS
HTA	52	107	0.85	(0.50–1.46)	0.38	NS
Tabac	55	109	0.88	(0.54–1.60)	0.88	NS
Diabète	29	61	0.23	(0.49–1.55)	0.23	NS
Obésité	23	45	0.91	(0.53–1.81)	0.01	NS
Hypercholestérolémie	43	87	0.91	(0.53–1.55)	0.14	NS
Siège Antérieur	66	135	0.80	(0.44–1.45)	0.63	NS

## Etude comparative

La fréquence des patients qui ont présenté une évolution compliquée au cours de leur hospitalisation a été significativement plus élevée dans le groupe des anémiés par rapport aux non anémiés. En effet les anémiés ont eu six fois plus de risque de faire une complication par rapport aux non anémiés. (86.6 vs 50.6%; OR = 6.34; IC 95%: 2.68–15.5; Khi^2^ = 23.56; *p* < 0.001).

Il y avait également une différence significative entre anémiés et non anémiés pour ce qui concerne l’évolution non compliquée et l’ensemble des complications et des décès. Les anémiés ont eu huit fois plus de risque d’avoir une évolution défavorable (complication ou décès) par rapport aux non anémiés (91.4 vs 57%; OR = 8.02; IC 95%: 3.5–19.07; Khi^2^ = 33.74; *p* < 0.0001).

L’insuffisance cardiaque gauche a été significativement plus élevée dans le groupe des anémiés par rapport aux non anémiés, tant pour les stades II et III de Killip que pour le choc cardiogénique (Killip IV): Les anémiés ont eu 3.7 fois plus de risque de présenter une insuffisance ventriculaire gauche (KILLIP II–III) (69.9% vs 38.5%; OR = 3.7; IC 95%: 2.08–6.60; Khi^2^ = 24.06; *p* < 0.0001); et six fois plus de risque pour le choc cardiogénique (24.7 vs 5.3%; OR = 6.21; IC 95%: 2.56–15.43; Khi^2^ = 22.89; *p* < 0.0001).

L’ensemble des troubles de l’excitabilité étaient supérieurs chez les anémiés par rapport aux non anémiés mais étaient à la limite de la significativité (40.8 vs 29.05%; OR = 1.69; IC 95%: 0.96–2.96; Khi2 = 3.86 *p* = 0.05). L’étude du détail des troubles du rythme n’a pas révélé de différence significative (Tableau 2).

**Tableau 2.** Comparaison Des Caracteristiques Cliniques Et Echographiques Des Anemies Et Des Non Anemies (Comparaison Of The Clinical And Echocardiographic Characteristics Of Anaemic And Non-Anaemic Patients)

*Variables*	*Anémiés (n = 93)*	*Non-anémiés (n = 179)*	*OR*	*IC 95%*	*Khi^2^*	*p*
Décès	33	23	3.73	1.94–7.19	19.18	0.00
IDM compliqué	52	79	6.3	2.68–15.5	23.5	0.00
Insuffisance VG (Killip II, III)	65	69	3.7	2.08–6.60	24.06	0.00
Choc cardiogénique	23	9	6.2	2.56–15.40	22.89	0.00
Troubles du rythme	38	52	1.7	0.96–2.96	3.86	0.05
Fibrillation auriculaire	4	6	1.3	0.29–5.42	–	NS
Extrasystoles auriculaires	11	9	2.5	0.92–7.02	4.15	0.04
Extrasystoles ventriculaires	22	37	1.19	0.62–2.27	0.32	NS
Tachycardie ventriculaire	3	3	1.96	0.30–12.6	–	NS
Fibrillation ventriculaire	3	5	1.16	0.21–5.80	–	NS
Troubles de la conduction	23	18	2.94	1.41–6.15	10.3	0.001
BAVI	6	3	4.05	0.86–21.1	–	NS
BAVII	5	2	5.03	0.83–38.8	–	0.05
BAVIII	6	2	6.10	1.07–45.3	–	0.02
BBD	10	6	3.52	1.12–11.4	6.18	0.01
BBG	2	2	1.97	0.19–20.2	–	NS
Fonction VG échographique						
FE < 40%	71	130	1.22	0.65–2.28	0.44	NS
%R < 28%	65	117	1.23	0.65–2.20	0.57	NS

VG = ventricule gauche

La fréquence des troubles de la conduction chez les anémiés a été 2.9 fois celle des non anémiés (24.7 vs 10%; OR = 2.94; IC 95%: 1.41–6.15; Khi^2^ = 10.3; *p* = 0.001). En réalité cette différence n’était significative que pour le BAV III (*p* = 0.02) et le bloc de branche droit (*p* = 0.012). Elle était limite pour le BAV II (*p* = 0.05). Les autres troubles de la conduction n’ont pas été significatifs: BAV I (*p* = 0.06), bloc de branche gauche (*p* = 0.41).

**Fig. 1.**
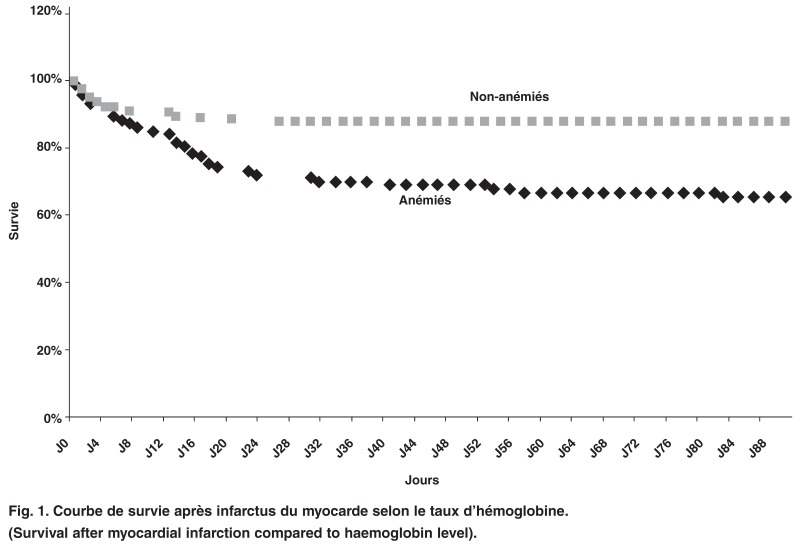
Courbe de survie après infarctus du myocarde selon le taux d’hémoglobine. (Survival after myocardial infarction compared to haemoglobin level).

La mortalité hospitalière des anémiés était 3.7 fois celle des non anémiés (35.5 vs 12,8%; OR = 3.73; IC 95%: 1.94–7.19; Khi_2_ = 19.18; *p* < 0.0001). Après un suivi de trois mois, la mortalité des anémiés a été supérieure à celle des non anémiés de 1.4 fois. Cette surmortalité commence dès l’admission et augmente progressivement comme le témoigne la courbe de survie à trois mois. Elle connaît une ascension particulière entre la première et la troisième semaine (Fig. 1).

Les facteurs de surmortalité intrahospitalière sont résumés dans le tableau 3. Ils étaient représentés essentiellement par le taux d’hémoglobine inférieur à 10 g/l (87%), le siège antérieur (69.6%), l’insuffisance ventriculaire gauche clinique (76.8%), la fraction d’éjection inférieure à 40% (92.8%).

**Tableau 3.** Caracteristiques Des Patients Decedes (Characteristics Of The Deceased Patients)

*Caracteristiques*	*%*
Age (ans)	55.7 ± 14 extrême: 22–90 ans
Sexe (M/F)	82/18
Tabagisme	39.3
HTA	37.5
Diabète	25
Dyslipidémie	16
Siège IDM	
Antérieur étendu	30.3
Circonférentiel	25
Antérieur	14.3
Insuffisance VG	76.8
Killip II (%)	17.9
Killip III (%)	19.6
Killip IV (%)	39.3
Troubles du rythme	41
Troubles de la conduction	21.4
Fraction d’éjection (< 40%)	93
Taux Hg < 10 g/l	87

Les anémiés décédés étaient plus âgés (59.2 ans vs 51.5 ans), avaient une durée moyenne d’hospitalisation plus longue (17.3 jours vs 6.2 jours) par rapport aux non anémiés. Ils étaient dans 20% des cas de sexe féminin contre 18.2% pour les non anémiés.

Les sujets âgés de 65 ans et plus étaient au nombre de 42 soit 15.4% de l’ensemble des deux populations. Parmi eux 22 (52.4%) étaient anémiés et 20 (47.6%) ne l’étaient pas. Le taux de mortalité parmi les anémiés était de 45.4% (10 patients) contre 10% chez les non anémiés (deux patients). Les 12 autres anémiés non décédés avaient tous présenté des complications hémodynamiques, rythmiques et ou conductives. Treize (65%) parmi les non anémiés ont présenté des complications. Aucun anémié âgé de 65 ans et plus n’a eu une évolution non compliquée. Par contre 25% des sujets âgés non anémiés ont eu une évolution non compliquée.

## Etude échographique

L’étude comparative de la fraction d’éjection du ventricule gauche n’a pas révélé de différence statistiquement significative entre anémiés et non anémiés (FE < 40%: 76.3 vs 72.6%, *p* = 0.44). Il en était de même pour la fraction de raccourcissement (*p* = 0.57)

## Discussion

L’anémie en particulier l’anémie carentielle constitue une pathologie fréquente en milieu tropical. Selon l’OMS5 le pourcentage de la population ayant moins de 10 g d’hémoglobine dépasse 30%. Elle est plus fréquente chez les enfants, les femmes enceintes et les sujets âgés.^5^,^6^ L’anémie occupe une place de choix parmi les patients hospitalisés à l’Institut de cardiologie d’Abidjan, et en particulier chez ceux atteints d’infarctus du myocarde. La moyenne du taux d’hémoglobine a été plus basse chez les anémiés que chez les témoins.

Les caractéristiques épidémiologiques de nos patients sont différentes de ceux de l’occident et d’Afrique du nord. En effet nos patients sont plus jeunes avec une moyenne d’âge nettement inférieure à celle de l’Europe et de l’Afrique du nord.^7^ L’hospitalisation est plus longue dans nos milieux qu’ailleurs. Les facteurs de risque les plus fréquemment associés à l’infarctus étaient le tabagisme et l’hypertension artérielle.

## Complications hémodynamiques

Plusieurs travaux ont mis en évidence le rôle péjoratif de l’anémie au cours des affections cardiovasculaires où elle est associée à une surmortalité. L’anémie provoquerait des perturbations hormonales et métaboliques qui ont un risque accru de toxicité myocardique, d’hypertrophie ventriculaire gauche et de rétention hydrosodée.^8^ Ainsi, la baisse de l’hématocrite sur un myocarde au préalable altéré par l’occlusion coronaire, accroît la nécrose avec comme conséquence immédiate l’insuffisance ventriculaire gauche. Notre étude soutient ces observations car comparativement aux témoins les patients anémiés ont eu plus de risque de faire une insuffisance ventriculaire gauche stade II ou III de KILLIP par rapport aux témoins (69.9 vs 38.5%; OR = 3.7; IC 95%: 2.08–6.60; *p* < 0.001).

Le risque de survenue de choc cardiogénique chez les patients anémiés était encore plus élevé (24.7 vs 5.3%; OR = 6.2; IC 95%: 2.56–15.43; *p* < 0.0001). Mais l’hypothèse du risque accru de choc cardiogénique chez les anémiés est controversée. Dauerman *et al*.^9^ dans leur série, ont conclu que le choc cardiogénique était plutôt en rapport avec les complications hémorragiques au cours de l’infarctus. Archbold^10^ dans une étude de cohorte ayant inclus 2 310 patients n’a pas trouvé de relation significative entre insuffisance ventriculaire gauche et taux d’hémoglobine à la phase aigue de l’infarctus du myocarde.

Néanmoins plusieurs autres auteurs ont mis en exergue le risque accru de survenue de dysfonction ventriculaire gauche à la phase aigue d’infarctus du myocarde chez les porteurs d’anémie sévère.^3^ La conséquence est l’augmentation de la mortalité chez les anémiés.

A l’échocardiographique par contre nous n’avions pas trouvé de différence statistiquement significative en ce qui concerne la fonction systolique du myocarde à la phase aiguë de l’infarctus entre anémiés et non anémiés (FE < 40%: 76.3 vs 72.6%, *p* = 0.44). Cette contradiction est due à la réalisation tardive des échographies chez nos patients. La plupart des échographies de notre série ont été réalisées en moyenne cinq jours après l’admission des patients où l’état hémodynamique de ces derniers s’était amélioré. Ceci est inhérent au caractère rétrospectif de notre étude.

## Les troubles du rythme

Le coronarien qui séjourne dans les milieux pauvres en oxygène notamment en altitude court un plus grand risque de présenter des troubles du rythme.^11^ L’hypoxie représente ainsi un facteur arythmogène. Au cours de l’infarctus du myocarde l’effet synergique de l’occlusion coronaire et de l’anémie aggrave l’hypoxie myocardique. Ainsi donc l’on observe une fréquence élevée de complications rythmiques à la phase aiguë de l’infarctus du myocarde chez les patients anémiés comme le témoignent nos résultats (40.8 vs 29.05%; OR = 1.69; IC 95%: 0.96–2.96; Khi^2^ = 3.86 *p* = 0.05)

## Les troubles de la conduction

Nous avons également observé une différence statistiquement significative entre les anémiés et les témoins en ce qui concerne les troubles conductifs. Les porteurs d’anémie ont eu plus de risque d’avoir un trouble conductif (tout type confondu) que les témoins (OR = 2.94; IC 95%: 1.41–6.15; *p* = 0.001). Mais en réalité la différence n’est significative que pour le BAV 3^e^ degré où le risque a été six fois plus élevé chez les anémiés que chez les témoins (*p* = 0.02), et le bloc de branche droit (*p* = 0.012).

La conséquence de toutes ces complications hémodynamiques, rythmiques et conductives est l’hospitalisation prolongée (21 jours vs 19.4 jours *p* = 0.03) et la surmortalité dans notre population globale singulièrement chez les anémiés.

## Mortalité

Le pronostic de l’infarctus du myocarde demeure encore sombre en Afrique tropicale avec 14% de décès à sa phase aigue.^12^ Nos résultats confirment cette surmortalité, en particulier chez les patients anémiés (35.5 vs 12.8%; *p* < 0.0001). Cet excès de surmortalité des anémiés au cours de l’infarctus du myocarde a été décrit par plusieurs auteurs. En effet Vaglio *et al.*^2^ dans une étude portant sur 1 038 patients, ont montré une mortalité à deux ans plus élevée chez les porteurs d’anémie sévère au cours de l’infarctus du myocarde. Ces résultats ont été confirmés par Valeur^3^ avec une mortalité accrue en cas d’anémie sévère et infarctus du myocarde. Arant^13^ a trouvé cette mortalité particulièrement plus élevée chez les femmes (10.3 vs 5.4%; *p* = 0.02).

Wu *et al*.^2^ ont quant à eux montré que de plus faibles taux d’hématocrite étaient associés à une surmortalité à 30 jours chez les sujets de plus de 65 ans. Cette mortalité avait atteint même les 50% en cas d’hématocrite inférieur à 27%. Parmi nos patients anémiés et âgés de 65 ans et plus, 45.5% sont décédés et 54.5% ont eu une complication intrahospitalière. Aucun d’entre eux n’a eu une évolution simple (*p* = 0.005). Ainsi donc les patients âgés de notre étude ont eu 100% de risque de faire une complication ou de décéder des suites immédiates de l’infarctus du myocarde. D’autres études ont confirmé non seulement la prévalence élevée de l’anémie, mais surtout la fréquence élevée de complications et de mortalité intrahospitalières au cours des syndromes coronariens aigus chez les sujets âgés anémiés, et ce malgré les gestes de revascularisation.^14^,^15^

La mortalité dans notre série était particulièrement liée à une association co-morbide. En effet les facteurs de surmortalité intrahospitalière étaient représentés essentiellement par le taux d’hémoglobine inférieur à 10 g/l (87%), le siège antérieur (69.6%), l’insuffisance ventriculaire gauche clinique (76.8%), la fraction d’éjection inférieure à 40% (92.8%), l’âge supérieur ou égal à 65 ans (45.4%). Le diabète retrouvé chez 33% de nos patients est reconnu comme facteur de surmortalité au cours de l’infarctus du myocarde,^7^ en particulier lorsqu’il est associé à l’anémie.^16^ L’effet co-morbide de ces facteurs associés à l’anémie dans une population où la thrombolyse a été rare, a eu comme conséquence une hospitalisation prolongée émaillée de complications avec in fine une augmentation de la mortalité intrahospitalière. Cette mortalité a connu une ascension particulière entre la première et la troisième semaine. Elle s’est poursuivi après trois mois de suivi, et est resté supérieure à celle des non anémiés de 1.4 fois (courbe de survie). Cette surmortalité a été mieux appréciée sur le long terme par d’autres auteurs. Elle est également significativement plus élevée chez les anémiés après un suivi médian de 24 mois.^17^,^18^

L’effet délétère de l’anémie sur le pronostic de l’infarctus du myocarde ne fait pas l’unanimité. En effet dans une étude réalisée par Al Falluji^4^ sur deux années, la mortalité à un an dans le groupe des patients souffrant d’anémie est superposable à celle du groupe des patients non anémiés. Dauerman^9^ va plus loin en concluant que la surmortalité post infarctus chez les anémiés était le fait des complications hémorragiques de l’anémie mais pas de l’anémie en elle même. Ces résultats contradictoires relancent le débat sur l’impact de l’anémie sur la mortalité post infarctus du myocarde. Nos résultats confortent la thèse du rôle péjoratif de l’anémie à la phase aigue de l’infarctus du myocarde.

Néanmoins Goodnough^19^ indique en accord avec Wu *et al*.^1^ que la transfusion serait salutaire en cas d’hématocrite inférieure ou égale à 33%. Mais il est difficile de définir le seuil à partir duquel la transfusion serait autorisée, car elle n’est toujours pas bénéfique pour le patient à la phase aigue de l’infarctus.^20^ La prudence est donc recommandée car la transfusion n’est pas bénéfique chez les patients qui ont un taux d’hématocrite supérieur à 33%.^1^

## Conclusion

Malgré la divergence des écoles, l’anémie apparaît de plus en plus dans la littérature actuelle comme un facteur important et déterminant du pronostic de l’infarctus du myocarde notamment à sa phase aiguë. Notre étude malgré la taille faible de son échantillon abonde dans cette hypothèse. L’association infarctus du myocarde aigu et anémie est source de mauvais pronostic car augmente de manière significative l’incidence de la dysfonction du ventricule gauche et la mortalité hospitalière à la phase aiguë de l’infarctus. Le risque est encore plus élevé chez les sujets âgés de 65 ans et plus.
